# Diagnostic discovery of structural variants causing foveal hypoplasia using SVRare and long-read nanopore sequencing

**DOI:** 10.1038/s41431-026-02190-y

**Published:** 2026-07-20

**Authors:** Mohammed A. M. Derar, Jing Yu, Christopher M. Watson, Martin McKibbin, Sandra M. Bell, Chris F. Inglehearn, Carmel Toomes

**Affiliations:** 1 Division of Molecular Medicine, Leeds Institute of Medical Research, Leeds, UK; 2 Nuffield Laboratory of Ophthalmology, Nuffield Department of Clinical Neurosciences, University of Oxford, John Radcliffe Hospital, Oxford, UK; 3 North East and Yorkshire Genomic Laboratory Hub, Central Lab, St. James’s University Hospital, Leeds, UK; 4 Department of Ophthalmology, St. James’s University Hospital, Leeds, UK

**Keywords:** Genetics research, Neurodevelopmental disorders

## Abstract

Advances in DNA sequencing technology are increasing the rate of molecular diagnosis for patients and families with inherited Mendelian diseases. However, some patients remain unsolved following standard-of-care testing (typically either exome or genome sequencing), with structural variants (SVs) likely to account for a significant proportion of these missed diagnoses. We re-analysed ten research cases with the visual disorder foveal hypoplasia (FH) that were previously unsolved through whole exome sequencing (WES). We used SVRare, a tool that integrates outputs from Canvas and Manta and combines these with allele frequencies and information regarding their genomic context to highlight plausible pathogenic SVs. This analysis solved 2/10 cases. Expanding this strategy, we then used SVRare in a reverse genetics approach to analyse an FH-relevant gene panel in the 100,000 Genomes Project (100KGP) rare disease cohort. This identified potentially pathogenic SVs as the cause of disease in a further 11 previously unsolved cases with phenotypes overlapping FH. In total, 11 SVs in five genes were identified as the likely cause of FH in 13 patients: *SLC38A8* (1 patient), *PAX6* (3), *OCA2* (3), *GPR143* (5) and *CACNA1F* (1). This analysis also identified multiple carriers of an apparently novel deletion in *OCA2*. However, further analysis suggested that this is, in fact, the relatively common founder variant responsible for oculocutaneous albinism (OCA) in African populations, first identified over 30 years ago. These findings show that SVRare effectively identifies deleterious SVs and illustrates the challenges in reporting SVs, including the importance of accurate and consistent reporting of such variants.

## Introduction

At the centre of the human retina lies the fovea centralis, the region of highest cone density, which is responsible for high acuity central vision under illuminated conditions [1]. The fovea is a depression in the retina which exposes the cone photoreceptors in the outer nuclear layer directly to incoming light. This specialised retinal structure reduces light diffraction from the inner retinal and ganglion cell layers that overlay photoreceptors elsewhere in the retina [2].

Genetic variants can cause defects in foveal development that compromise the structure and depth of the foveal pit, resulting in a condition of reduced visual acuity known as foveal hypoplasia (FH). The hallmarks of FH include the presence of the inner retinal layers and vasculature at the foveal region, which can be identified using optical coherence tomography (OCT) and fundoscopy, respectively [3]. FH is frequently observed as part of the clinical spectrum in a range of eye diseases encompassing albinism, aniridia, optic nerve hypoplasia, achromatopsia, Stickler syndrome, incomplete congenital stationary night blindness (CSNB), incontinentia pigmenti, familial exudative vitreoretinopathy and nanophthalmos. FH, in association with nystagmus and chiasmal misrouting, can also present in the absence of other symptoms, as is seen in foveal hypoplasia 2 (FVH2) due to *SLC38A8* variants [4]. There is significant phenotypic overlap between FH and other inherited retinal conditions (IRDs), especially where there is variable expressivity. Consequently, a differential diagnosis of FH is often obtained through genetic assessment [5]. To date, pathogenic variants in more than 30 different genes have been shown to cause FH, with autosomal recessive, autosomal dominant and X-linked inheritance [5, 6].

The most common class of genetic variants reportedly causing human disease is single nucleotide variants (SNVs), followed by short insertions or deletions (INDELs) and, less frequently, larger structural variants (SVs). The latter represents a class of variants that affect at least 50 nucleotides and can include deletions, insertions, duplications, inversions and translocations [7]. SVs are potentially underreported as they are more difficult to detect, due to inherent limitations associated with the assembly of short-read sequences. Repetitive sequences, which are often present at SV breakpoints, can result in the ambiguous mapping of sequence reads, and other factors such as high Guanine-Cytosine (GC) content can reduce coverage at the target site [8]. SVs therefore pose a significant diagnostic challenge and are estimated to account for ~11% of undiagnosed cases of IRD [9]. Dedicated computational tools are required to identify SVs. Typically, this involves assessing a relative change in sequence read coverage, indicative of a copy number variant (CNV) [10] and the detection of discordant read pairs arising from aberrant alignments, which may point to inversion or translocation events [11]. The prioritisation of SVs identified by different variant calling tools can be achieved using SVRare, which offers a unified approach and a comprehensive annotation of aggregated SVs [12]. SVRare annotations also include the allele frequency data in the supplied cohort and the genomic context, in order to highlight affected genes and the potential disruptions to the coding sequence (CDS) [12, 13].

The UK 100,000 genomes project (100KGP) [14] is a national whole genome sequencing (WGS) initiative led by NHS England, which recruited 72,934 participants from families with rare disease and 17,239 cancer patients. The genomic data from these patients has been made available to researchers through the Genomics England Research Environment to facilitate the identification of potential genomic diagnoses and accelerate the translation of genomic discoveries into clinical practice (https://re-docs.genomicsengland.co.uk/release19/). At the time this study was initiated, variants ≥50 bp were not detected or analysed by the 100KGP variant prioritisation and annotation pipelines.

In this study, we evaluate the utility of SVRare for identifying pathogenic SVs in a research cohort of FH patients who remained unsolved after whole exome sequencing (WES), and in individuals with inherited retinal disease recruited to the 100KGP. SVs identified by SVRare in the research cohort were further evaluated using long-read sequencing. In total, 11 SVs were identified as the likely cause of FH in 13 patients. The implicated genes include *SLC38A8* (1 patient), *PAX6* (3), *OCA2* (3), *GPR143* (5) and *CACNA1F* (1). SVRare analysis of WGS, combined with long-read nanopore sequencing, has significantly improved the diagnostic yield in FH and demonstrated the value of SVRare for the interpretation of SVs.

## Materials and methods

### Patient recruitment and assessment

Probands and their family members were recruited through either hospitals with eye clinics or the UK 100KGP. Informed and written consent was obtained in accordance with the principles of the Declaration of Helsinki. Probands either had a confirmed diagnosis of FH or presented with a phenotype of suspected FH. Ethical approval for this project was granted by the Leeds East NHS Research Ethics Committee (Project number 17/YH/0032). The 100KGP is covered by REC reference approval 14/EE/1112. Genomic DNA from locally recruited cases was isolated from peripheral blood lymphocytes by the North-East and Yorkshire Genomics Laboratory Hub using a bead-based extraction method. Medical history was obtained, and where possible, family members underwent ophthalmic assessment, including fundus examination and OCT.

### Short read WGS and analysis

Approximately 1.5 µg of genomic DNA (50–100 ng/µl) in a minimum volume of 30 µl was sent to Edinburgh Genomics (University of Edinburgh) for whole genome sequencing. DNA libraries from local cases were prepared using the SeqLab TruSeq Nano DNA Library Prep HT kit (Illumina) following the manufacturer’s instructions. Sequencing was performed on a HiSeq X (Illumina, San Diego, California, USA) using v.2.5 reagents and flow cells to generate paired end 150 bp reads. Raw data were converted to FASTQ format before sequence reads were aligned to the human reference genome (either build GRCh37 or GRCh38) using the Burrows Wheeler Aligner (BWA-MEM) v.0.7.13 [15]. Duplicate reads were marked using SAMBLASTER (v.0.1.22) [16] and variant calling of SNVs and INDELs was performed using the HaplotypeCaller v3.5 following Genome Analysis Toolkit (GATK) best practice guidelines [17].

### SV discovery by SVRare in a research cohort

Probands from the research cohort who presented with FH but without any evidence of a pigmentary defect, and who were unsolved following analysis by WES, were reanalysed by WGS. CNVs identified using Canvas and SVs detected using Manta were collated and annotated by SVRare [12]. SVRare software can be obtained from https://github.com/Oxford-Eye/SVRare-db/tree/master, with an additional front-end visualisation app code available via https://github.com/Oxford-Eye/SVRare-js. Identified variants were retained if their allele frequency was < 3 in an in-house cohort of 374 exome and 96 genome sequences from individuals with IRDs and they were absent from the Genome Aggregation Database (gnomAD) SVs database v.4.1.0 (https://gnomad.broadinstitute.org/news/2023-11-v4-structural-variants/), the NCBI Database of human genomic structural variation (dbVAR https://www.ncbi.nlm.nih.gov/dbvar/) and the DECIPHER database (https://www.deciphergenomics.org/). SVs were prioritised if they disrupted the coding sequence of genes implicated in FH. Around 30 such candidate SVs were manually verified per genome by inspecting sequence reads in the Binary Alignment Map (BAM) file using the Integrative Genomics Viewer (IGV) v.2.8.13 [18]. SVs were reported according to Human Genome Variation Society (HGVS) nomenclature and classified using the American College of Medical Genetics (ACMG) guidelines by the Franklin webserver (https://franklin.genoox.com/clinical-db/home).

### Long-read sequencing and bioinformatic analysis

Primers were designed using Primer3Web (http://primer3.ut.ee/) before being validated using PrimerBLAST (https://www.ncbi.nlm.nih.gov/tools/primer-blast/) and SNPCheck V3 (https://genetools.org/SNPCheck/snpcheck.htm). Amplification of PCR targets was achieved using the SequalPrep^TM^ Long PCR Kit with dNTPs (Invitrogen) according to the manufacturer’s instructions. Thermocycler parameters were an initial denaturation at 94 °C for 2 min, then 10 cycles of 94°C for 10 s, 56–58 °C (*PAX6* variant) or 55–60 °C (*GPR143* variant) for 30 s and 68 °C for 1 min/kb, followed by 30 cycles of 94 °C for 10 s, 50–65 °C for 30 s and 68 °C for 1 min/kb (+20 s per cycle). A final extension was performed at 72 °C for 5 min. PCR amplification products were cleaned up using the AMPure XP reagent (Beckman Coulter). Nucleic acid quantification was carried out using the Qubit^TM^ dsDNA BR Assay Kit (Invitrogen). Amplification products were pooled at equimolar concentration to give a total of 100 fmol in 23.5 μl. DNA libraries were prepared using Oxford Nanopore Technologies (ONT) Ligation Sequencing Kit (SQK-LSK110) according to the manufacturer’s instructions and sequencing was performed on a MinION Mk1B (ONT, Oxford, UK) using a Flongle flow cell (R9.4.1) that was run for 24 h. The raw signal was captured using the MinION control software minKNOW v.21.11.7 (ONT). Base-calling was performed using Guppy v.5 (https://nanoporetech.com/). Adaptors were trimmed from the reads using Porechop v.0.2.4 (https://github.com/rrwick/Porechop) before reads were filtered by length (75-3000 bp) and quality (≥Q10) using NanoFilt v.2.8.0 (https://github.com/wdecoster/nanofilt). Read metrics were obtained using NanoStat (https://github.com/wdecoster/nanostat). Sequence reads were aligned to the human reference genome (either GRCh37 or GRCh38) using Minimap2 v.2.22 (https://github.com/lh3/minimap2) [19]. Samtools v.1.9 (https://github.com/samtools/samtools) was used for the conversion of Sequence Alignment Map (SAM) to BAM format and the sorting of reads by genomic coordinate. The resulting indexed BAM files were visualised using IGV v.2.8.13.

### Gene-specific analysis of SVs in the 100KGP

Participant data from individuals recruited to the UK 100KGP was accessed as part of project 582, Gene and Variant Discovery in inherited eye diseases, using the secure Genomics England research environment (https://re.extge.co.uk/ovd/). The reported phenotype, in Human Phenotype Ontology (HPO) format, was obtained from Participant Explorer v.5.6.0. Participants’ solved status was obtained from the GMC exit questionnaire provided in Labkey v.23.7. SVRare compiled 554,060,126 SVs called by Manta and Canvas in the 71,408 participants with rare disease [12]. These were filtered to identify those affecting 28 genes implicated in aniridia, albinism, Hermansky-Pudlak syndrome, Chediak-Higashi syndrome, Stickler syndrome, incomplete congenital stationary night blindness (CSNB) and isolated FH. The list of genes included is based on those reviewed in [5], with some modifications: *AHR*, *AP3B1*, *AP3D1*, *BLOC1S3*, *BLOC1S5*, *BLOC1S6*, *CACNA1F*, *COL11A1*, *COL2A1*, *DCT*, *DTNBP1*, *FRMD7*, *GPR143*, *HPS1*, *HPS3*, *HPS4*, *HPS5*, *HPS6*, *IKBKG*, *LRMDA*, *LYST*, *OCA2*, *PAX6*, *SLC24A5*, *SLC38A8*, *SLC45A2*, *TYR* and *TYRP1*. Allele frequencies derived from SVRare were compared to the gnomAD SVs database v.4.1.0 (https://gnomad.broadinstitute.org/news/2023-11-v4-structural-variants/). SVs identified in participants who had already received a genomic diagnosis through the 100KGP were excluded and the remaining SVs were retained if they had a recurrence rate <= 1% among all GEL participants. SVs were then verified to discard false-positive calls through manual inspection of the participant’s BAM file using IGV v.2.15.4. ACMG classification was determined using the Franklin web server (https://franklin.genoox.com/clinical-db/home). The SVs were then analysed to determine whether, either alone or in combination with other known variants, they could explain the participant’s phenotype.

### Haplotype analysis in 100KGP participants

Haplotype analysis was performed on participants carrying an SV in *OCA2*. The genomic Variant Call Format (VCF) file for each participant and their parents was located using the genome_file_paths_and_types link on LabKey v.23.7. The locus Chr15:27979889-28024696 (GRCh38) was examined using IGV v.2.15.4 for each participant. The unique identifier assigned to each variant is based on the dbSNP identification system (https://www.ncbi.nlm.nih.gov/snp/). Variants with a confidence score ≥79 QUAL, implying a high probability that the variant exists at the reported locus, were retained. Genotypes were verified using the participants’ BAM files to eliminate artefacts. Phasing of alleles in probands was determined by comparison with parental genotypes. Validation of the phased haplotype was achieved using ONT long-read sequencing in one participant. Allele frequencies in African and European populations were derived from gnomAD v.4.1.0 (https://gnomad.broadinstitute.org/).

## Results

A cohort of ten FH patients without reported hypopigmentation, who remained unsolved after WGS, was analysed. SNVs and INDELs were called using the GATK HaplotypeCaller, CNVs detected using Canvas and SVs identified by Manta were collated and annotated using SVRare.

### Identification of a heterozygous *PAX6* deletion

**Fig. 1:**
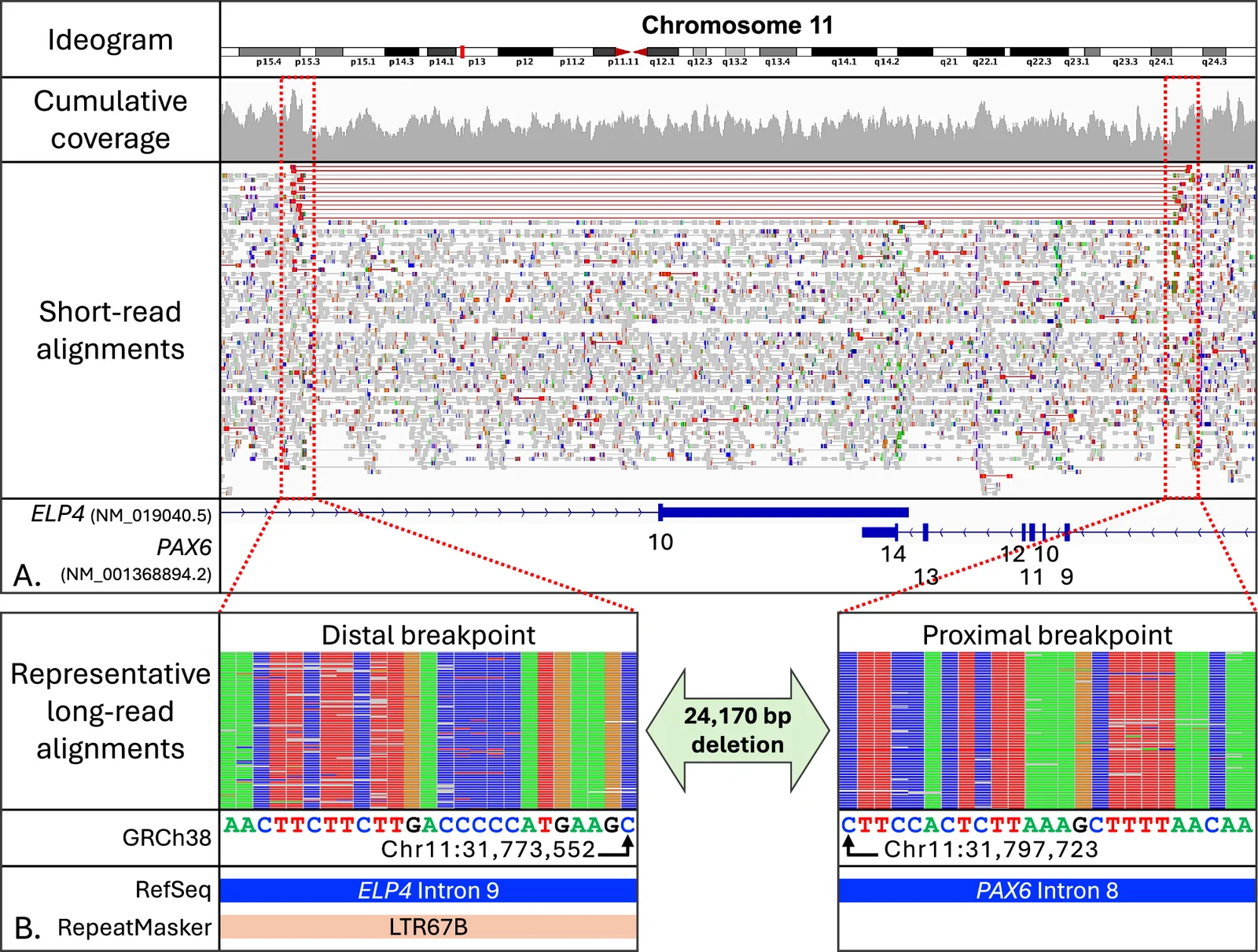
Characterisation of CNV in proband P1. **A** IGV plot of short-read sequencing alignments showing the *PAX6*/*ELP4* locus. Read pairs with a larger-than-expected insert are displayed in red and provide evidence for a deletion variant. Consistent with this, the cumulative coverage plot displays a reduction in sequence read depth across the deleted region, indicating that it is a heterozygous deletion. Exons are numbered according to the specified transcripts for each gene and arrows show the direction of transcription. **B** IGV plot of nanopore long-read sequences showing the nucleotide sequence at the deletion breakpoint. This analysis was based on down-sampling from over 16,000 reads. Genomic coordinates are provided according to build GRCh38 of the human reference genome.

European proband P1 presented with isolated FH in the absence of any previous family history or other symptoms. No further clinical data are available for this case. SVRare analysis revealed a heterozygous deletion spanning 24.2 kb on chromosome 11 (Fig. 1A). The deletion, which SVRare defines as NC_000011.10:g.31773553_31797722del, encompasses exons 9-14 of *PAX6* (NM_001368894.2) and exon 10 of *ELP*4 (NM_019040.5) and is absent from gnomAD SV, dbVAR and DECIPHER. It is notable that the distal breakpoint intersects an LTR67B long terminal repeat element. The SV was classified as pathogenic according to ACMG criteria. Variants in the *PAX6* gene are a known cause of autosomal dominant FH, as well as aniridia and other forms of anterior segment dysgenesis [20, 21], and large SVs are frequently documented as a cause of disease at this locus [22]. Long-range PCR enrichment in combination with long-read nanopore sequencing was used to verify the deletion breakpoint (Fig. 1B).

### Identification of a hemizygous *GPR143* deletion

**Fig. 2:**
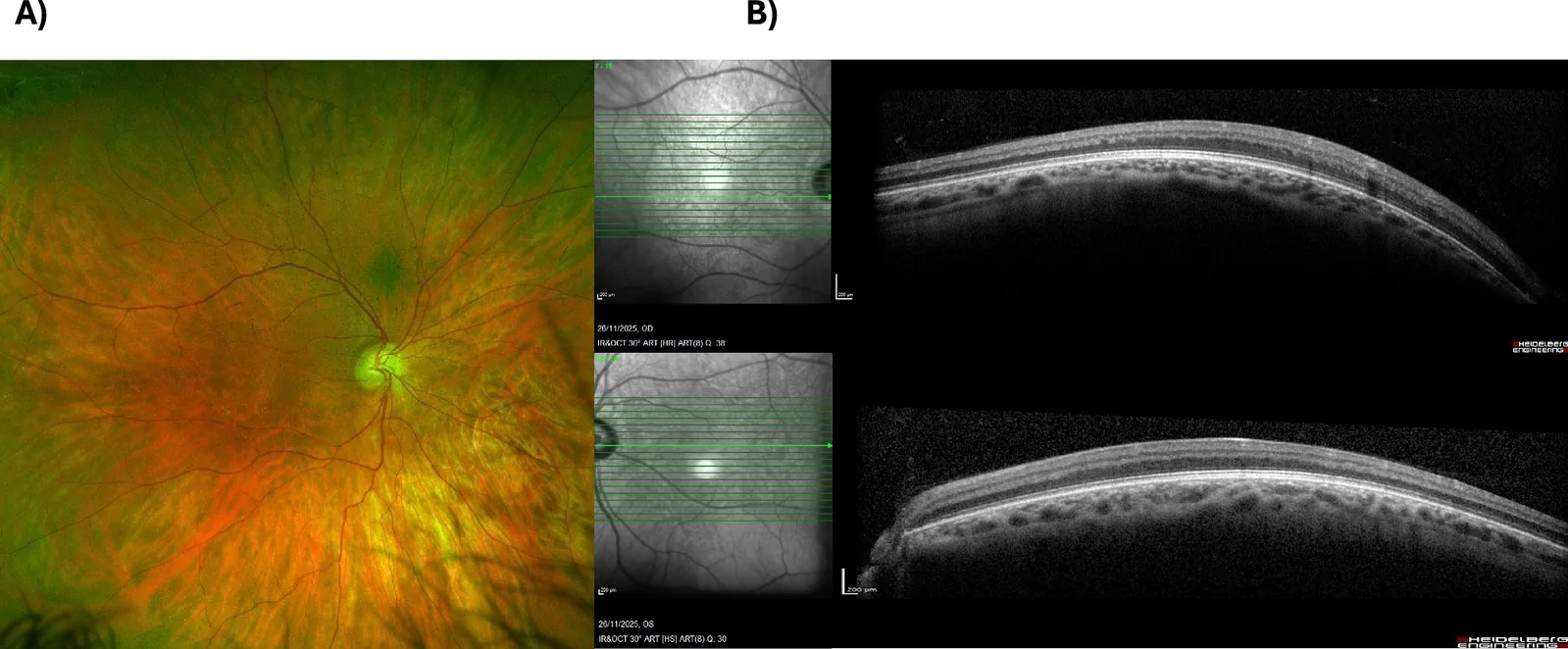
Phenotype observed in proband P2. **A** Colour fundus image of the right eye from proband P2, showing a tessellated fundus with prominent choroidal blood vessels, consistent with ocular albinism. **B** OCT infrared reflectance and line scan images of the right (upper) and left (lower) eyes from proband P2. Retinal lamination appears normal, but the foveal pit is absent. There is a dome-shaped configuration to the macula in both eyes.

**Fig. 3:**
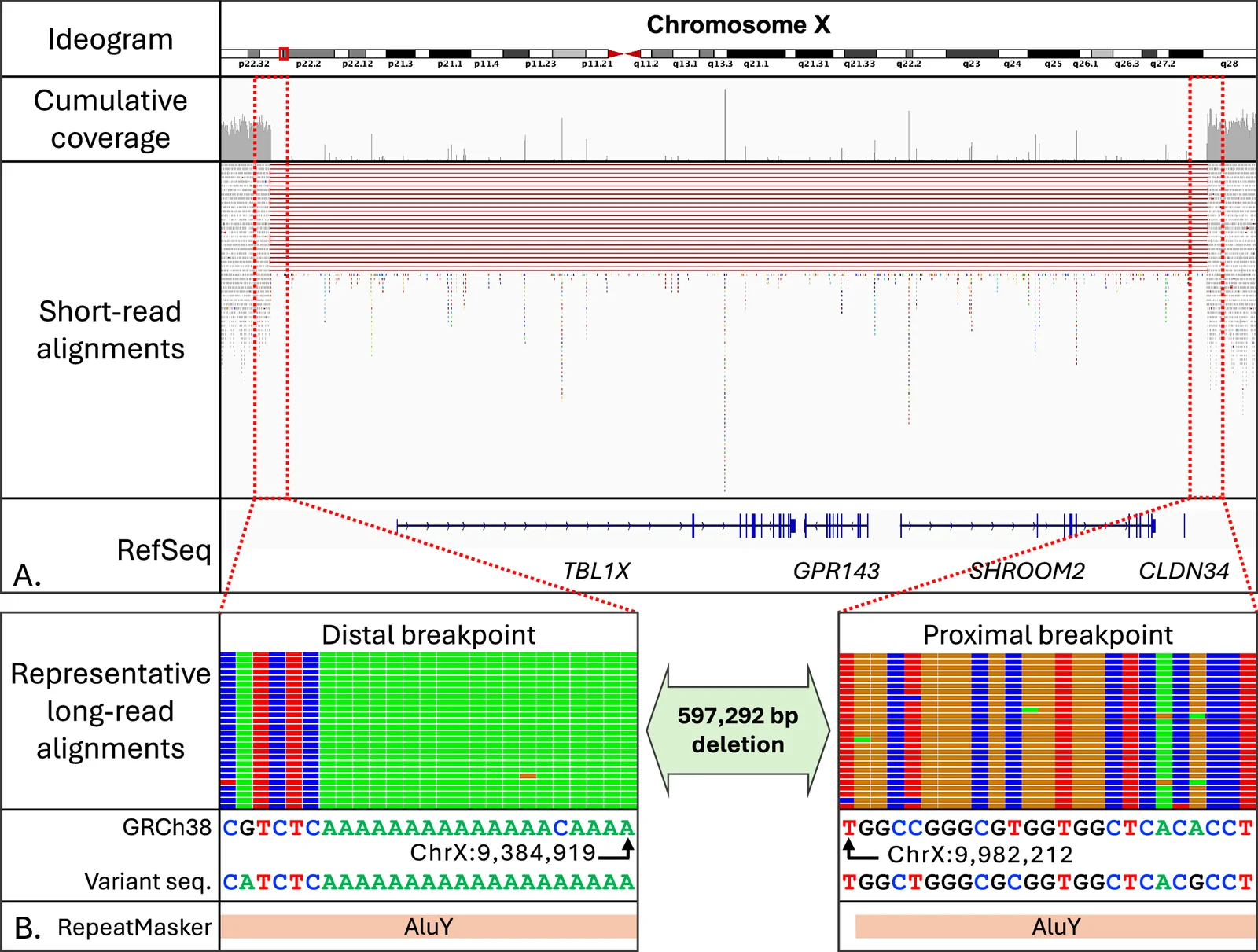
Characterisation of CNV in proband P2. **A** IGV plot of short-read sequencing alignments displays an almost complete absence of reads mapping to *TBL1X*, *GPR143*, *SHROOM2*, and *CLDN34* genes. The small number of reads that do align within the deleted region are spurious low-quality alignments that map to low-complexity repeat sequences. Discordant primer pairs connected by a red line indicate an alignment insert larger than the expected size and provide support for a deletion variant. The cumulative coverage plot further supports the deletion event. **B** IGV plot of representative nanopore long-read sequence at the deletion breakpoints which intersect AluY SINE elements. This analysis was based on down-sampling from over 49,000 reads. Genomic coordinates are provided according to build GRCh38 of the human reference genome.

In a South Asian family, proband P2 presented with poor visual acuity, nystagmus and a dome-shaped configuration of the macula in both eyes, consistent with ocular albinism. Colour fundus imaging of the right eye showed a tessellated fundus with prominent choroidal blood vessels (Fig. 2). The patient did not present with cutaneous hypopigmentation and there was no report of an iris transillumination defect (TID). SVRare analysis annotated multiple SVs, including a CNV encompassing *GPR143*. The male proband was found to be hemizygous for a deletion of 597.3 kb on the X chromosome and the approximate cumulative read depth at the deletion boundary was 35× (Fig. 3A). The deletion removes four genes in their entirety, *TBL1X*, *GPR143*, *SHROOM2* and *CLDN34*. The variant was classified as pathogenic by ACMG criteria, being absent from gnomAD SV, dbVAR and DECIPHER. Pathogenic *GPR143* variants are a known cause of X-linked ocular albinism (OA) [23], making this variant a likely cause of FH in this patient. The deletion breakpoints were poorly resolved by short-read sequencing, prompting further analysis by long-read nanopore sequencing. Both breakpoints intersected AluY short interspersed nuclear elements (SINEs), making their precise characterisation challenging; nevertheless, the consensus nomenclature was estimated to be NC_000023.11: g.9384920_9982211del (Fig. 3B).

### Detecting SVs in 100KGP participants with FH

Participants in the 100KGP were recruited through busy NHS clinics and often reported with only partial information on the clinical manifestations [24]. To compensate for the possibility of incomplete reporting of phenotypes, or the use of HPO terms not specific for FH, a gene-specific analysis was performed on all 100KGP participants irrespective of the reported phenotype. This reverse genetics approach was applied to screen for SVs in 28 FH-implicated genes using SVRare.

In total, 2256 SVs were identified in 28 genes, distributed across all 71,408 100KGP participants with rare disease. The variants were filtered based on allele count and potential disruption to the coding sequence, resulting in the prioritisation of nine unique SVs in 11 participants who had not previously received a molecular diagnosis (Table 1; Supplementary fig. S1). The SVs identified included an intragenic *SLC38A8* inversion and deletions in *PAX6*, *OCA2*, *GPR143* and *CACNA1F*. Typically, variants were unique to a single individual, except for an insertion-deletion in *OCA2* that was identified in participants P4 and P5 and a deletion in *GPR143* that was identified in participants P6 (the proband) and P7 (P6’s maternal grandfather). As expected, the *GPR143* deletion was also detected in the proband’s mother. All variants reported in the 11 participants are potentially disease-causing, either alone or in combination with other potentially pathogenic variants already identified. Cumulative read depth at the variant locus for each SV ranged from 31-78×, and the reported HPO terms for each individual suggested a retinal defect that is compatible with an FH phenotype. The SVs are therefore likely to account for the disease identified in each of the individuals, and the genomic diagnoses were relayed to the recruiting clinicians via 100KGP clinical collaboration requests.

**Table 1:** Likely pathogenic structural variants and clinical findings in previously unsolved 100KGP participants with diagnoses consistent with foveal hypoplasia.

ID	Phenotype	Gene	Genotype	Class	SV	Effect	100KGP	gnomAD	Gender	ACMG
P3	Retinal dystrophy, central scotoma, visual impairment, nonprogressive visual loss and reduced visual acuity	*OCA2*	Homozygous	Deletion	NC_000015.10: g.27823041_27849013del (25973 bp)	Out-of-frame deletion of exon 23	2/71408	0	F	Likely pathogenic
P4	Nonprogressive visual loss, central scotoma, visual impairment, macular atrophy and reduced visual acuity	*OCA2*	Compound heterozygous	Deletion	NC_000015.10: g.28017720_28020677delinsTTT (2958 bp)	Out-of-frame deletion of exon 7	30/71408	0	F	Pathogenic
				Missense	c.2228 C > T, p.(Pro743Leu)	–	–	287/1613590		Pathogenic
P5	Visual impairment and foveal hypoplasia	*OCA2*	Compound Heterozygous	Deletion	NC_000015.10: g.28017720_28020677delinsTTT (2958 bp)	Out-of-frame deletion of exon 7	30/71408	0	M	Pathogenic
				Missense	c.2323 G > C, p.(Gly775Arg)	–	–	6/1613792		Pathogenic
P6*	Nonprogressive visual loss, retinal dystrophy, visual impairment, central scotoma and macular atrophy	*GPR143*	Hemizygous	Deletion	NC_000023.11 :g.9476905_9954651del (477747 bp)	Gene deletion	14/71408	0	M	Pathogenic
P7*	Nonprogressive visual loss, retinal dystrophy, reduced visual acuity, central scotoma and visual impairment	*GPR143*	Hemizygous	Deletion	NC_000023.11 :9476905_9954651del (477747 bp)	Gene deletion	14/71408	0	M	Pathogenic
P8	Visual impairment, retinal dystrophy, reduced visual acuity, central scotoma and nonprogressive visual loss	*GPR143*	Hemizygous	Deletion	NC_000023.11 :g.9743119_10024473del (281355 bp)	Deletion of exons 1-6	1/71408	0	M	Likely pathogenic
P9	Visual impairment, retinal dystrophy, central scotoma and nonprogressive visual loss	*GPR143*	Hemizygous	Deletion	NC_000023.11: g.9760253_9761254del (1002 bp)	Out-of-frame deletion of exon 2	1/71408	0	M	Likely pathogenic
P10	Aniridia	*PAX6*	Heterozygous	Deletion	NC_000011.10 :g31393557_31812922del	Gene deletion	1/71408	0	M	Pathogenic
P11	Aniridia	*PAX6*	Heterozygous	Deletion	NC_000011.10 :g.28833790_37084393del (8250604 bp)	Gene deletion	1/71408	0	F	Pathogenic
P12	Nystagmus and visual impairment	*SLC38A8*	Homozygous	Inversion	NC_000016.10 :g.84015931_84027116inv (11186 bp)	Inversion of exons 7-9	1/71408	0	F	Pathogenic
P13	Visual impairment, abnormal light-adapted electroretinogram, non-progressive visual loss and reduced visual acuity	*CACNA1F*	Hemizygous	Deletion	NC_000023.11 :g.49224054_49226365del (2312 bp)	In-frame deletion of exons 12-14	2/71408	0	M	VUS

Individuals P6 and P7 are related (proband and maternal grandfather, respectively), denoted by a *.

### Identification of a frequently encountered SV in *OCA2*

Amongst the SVs identified in 100KGP participants with potential FH, an insertion-deletion in *OCA2* defined by SVRare as NC_000015.10: g.28017720_28020677delinsTTT was present in two apparently unrelated probands of African descent. Proband P4 also inherited a second pathogenic variant in *OCA2* (ClinVar ID: VCV000000956.37), NM_000275.3:c.2228 C > T, NP_000266.2:p.(Pro743Leu), Chr15(GRCh38):g.27871170 G > A, which could potentially be arranged in *trans* (present on the other parental allele). However, as parental specimens or parental genomic data were not available, the allelic phase could not be established. The missense variant is predicted to be deleterious by CADD v1.7 (score: 31), SIFT v.6.2.1 (score: 0) and PolyPhen-2 v.2.2.3 (0.985). For proband P5, the recorded HPO terms were visual impairment and FH. This patient had also inherited a second *OCA2* pathogenic variant (ClinVar ID: VCV001460031.9), NM_000275.3:c.2323 G > C, NP_000266.2:p. (Gly775Arg), Chr15(GRCh38):g.27851397 C > G that may be arranged in *trans*. Similarly to case P4, phasing was not possible as no parental specimens or parental genomic data were available. In silico pathogenicity assessment of the NP_000266.2:p.(Gly775Arg) variant generated scores for CADD v1.7 (26.4), SIFT v.6.2.1 (0) and PolyPhen-2 v.2.2.3 (0.97), indicating it is likely to be damaging.

Interestingly, in addition to individuals P4 and P5, the *OCA2* CNV was identified in a further 28 participants in the 100KGP. These participants did not have a second potentially pathogenic *OCA2* variant and had phenotypes unrelated to FH. Carrier status for the NC_000015.10: g.28017720_28020677delinsTTT variant was confirmed in all 30 participants following manual inspection of the aligned sequence reads, which verified the deletion as heterozygous in each case. The 30 participants include 18 individuals of African or Afro-Caribbean ethnicity (60%), 6 of mixed African or mixed Afro-Caribbean ethnicity (20%), 2 of other ethnicities (6.7%) and 4 of undisclosed ethnicity (13.3%). Of these *OCA2* SV carriers, at least 80% are therefore of African or of mixed African ancestry, despite this demographic representing only 3.1% (2767/90173) of the entire 100KGP population. Significant enrichment of the *OCA2* insertion-deletion in probands of African or Afro-Caribbean ethnicity suggests that this may be a founder allele that originated in individuals of African descent. Haplotype analysis was performed to test this hypothesis.

Three participants of African or Afro-Caribbean ethnicity, and for whom full trio data was available, were selected as representative cases (denoted carrier 1, 2 and 3). Haplotypes for the *OCA2* insertion-deletion-containing alleles were constructed across a 44,808 bp interval (Chr15:27979889-28024696), with single nucleotide markers being phased based on their pattern of parental inheritance (Supplementary Table S1). This analysis revealed a shared haplotype that encompassed the NC_000015.10: g.28017720_28020677delinsTTT variant by all three carriers across the entire 44.8 kb region.

**Fig. 4:**
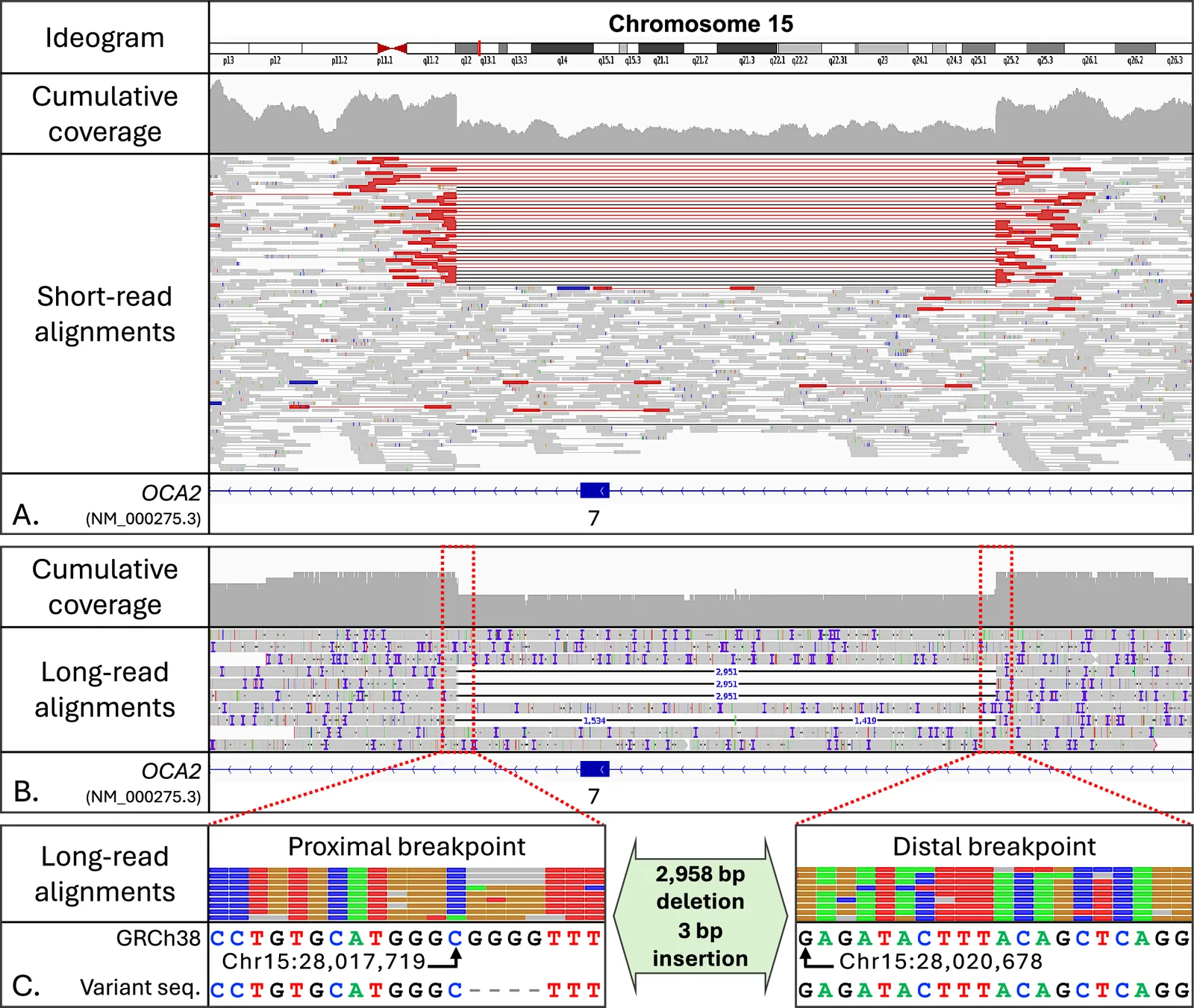
Characteristics of the recurrent *OCA2* insertion-deletion variant NC_000015.10: g.28017720_28020677delinsTTT in a 100KGP participant. **A** IGV plot of short-read WGS data for carrier 4. Read-pairs highlighted in red indicate the insert is larger than expected and provide support for the deletion event. The cumulative coverage plot displays a visible reduction in sequence reads across the deleted region. **B** An IGV plot of long-read nanopore alignments at the deletion-containing locus; gapped alignments show the extent of the deleted region. This analysis was based on the 15 reads that were available. **C** Long-read alignments at the deletion breakpoints enable characterisation of the insertion-deletion variant at nucleotide resolution. Genomic coordinates are provided according to build GRCh38 of the human reference genome.

Furthermore, both short-read and long-read nanopore sequencing data were available through the 100KGP for a fourth Afro-Caribbean individual, carrier 4, who is also heterozygous for NC_000015.10: g.28017720_28020677delinsTTT (Fig. 4A, B). This data enabled orthogonal verification of the variant. Within the region Chr15:27979889-28024696 (GRCh38) encompassing the insertion-deletion variant (Fig. 4C). Long-read WGS supported a 24,699 bp haplotype that matched the haplotype obtained from three carriers via short-read NGS, confirming the shared Afro-Caribbean haplotype encompassing the SV (Table 2).

**Table 2:** Haplotype at the *OCA2* locus in carriers of OCA2 NC_000015.10: g.28017720_28020677delinsTTT.

The red box defines the haplotype confirmed by long-read sequencing in carrier 4.

## Discussion

Advances in DNA sequencing technologies over the past 30 years have enabled a considerable increase in our understanding of the aetiology of inherited human diseases, allowing accurate molecular diagnoses and informed genetic counselling for many families. Nevertheless, molecular testing results in a proportion of cases remaining unsolved, with SVs that are refractory to the initial analysis likely to account for many of these missed diagnoses. In this study, we reanalysed ten research cases with FH and a cohort from the 100KGP with phenotypes overlapping with FH. These were screened using SVRare, a recently published [12, 13] tool that integrates outputs from Canvas and Manta and combines these with allele frequencies and the genomic context to highlight plausible pathogenic SVs. The approach has led to a significant increase in diagnostic success, solving two out of ten FH research cases, consistent with a previous estimate for the proportion of inherited diseases caused by SVs [9], and eleven previously unsolved cases in the 100KGP cohort.

SVs identified in the FH research cohort were further characterised using long-range PCR target enrichment in combination with long-read nanopore sequencing, using a previously described workflow [25]. In proband P1, SVRare analysis identified deletion of exons 9-14 of *PAX6* and of exon 10 of *ELP4*. Variants in *PAX6* are a well-documented cause of FH [20, 21]. Isolated FH is more commonly attributed to missense variants [26], while SVs are more often associated with the more severe phenotype of aniridia, a malformation of the iris. Indeed, a recent study identified two *PAX6* deletion variants that also encompass exons 9-14 of *PAX6* and exon 10 of *ELP4*, NC_000011.10:g.31777022_31796016del and NC_000011.10:g.31780738_31796994del, both of which resulted in aniridia [27]. The absence of aniridia-associated features such as iris hypoplasia or coloboma in proband P1 is therefore notable. Nevertheless, the identification of NC_000011.10: g.31773553_31797722del in this study expands the *PAX6* mutation spectrum.

WGS of proband P2 revealed a deletion of four genes, *TBL1X*, *GPR143*, *SHROOM2* and *CLDN34*, with the deletion of *GPR143* being the likely cause of FH [23]. This was again confirmed by long-read sequencing, which supported the breakpoints ChrX:9384920-9982211. Hemizygosity of *GPR143* pathogenic variants is typically associated with OA [23], which presents with FH, TID, fundus hypopigmentation and nystagmus, but proband P2 was diagnosed only with FH and nystagmus. This could be due to variability of phenotypic expression with age, which has previously been reported in albinism [28]. Furthermore, OA patients with the same pathogenic *GPR143* genotypes have been reported to show variability in the presentation and severity of iris TID, fundus hypopigmentation and nystagmus [29].

The 100KGP cohort had not been systematically screened for SVs prior to analysis using SVRare. SVRare analysis of 28 genes linked to FH has identified ten pathogenic or likely pathogenic SVs and one SV classified as a variant of uncertain significance (VUS), in previously unsolved cases with phenotypes overlapping FH. These were found in three individuals with oculocutaneous albinism (OCA) due to SVs in *OCA2* (P3-P5), four with OA due to variants in *GPR143* (P6-P9), two with aniridia caused by *PAX6* SVs (P10-P11), one with FH due to an SV in *SLC38A8* (P12) and one with incomplete CSNB due to a *CACNA1F* SV (P13). None of these SVs are listed in the gnomAD SV database, even though one, NC_000015.10: g.28017720_28020677delinsTTT, was present in 30 100KGP participants. GnomAD SV v4 currently catalogues 1,199,117 SVs, a relatively low number when compared to the 786,500,648 SNVs and 122,583,462 INDELs listed (https://gnomad.broadinstitute.org/stats, visited 25/05/25). This discrepancy likely reflects the difficulty in detecting SVs using current and legacy sequencing technologies. Furthermore, short-read sequencing often fails to resolve SV breakpoints, resulting in variable reporting of the same variant. Long-read sequencing can help to overcome these limitations by providing more accurate characterisation of specific variants, especially in intractable genomic regions [30]. We have previously reported the reliable use of this technology in a separate study [25]. More accurate characterisation of SVs would ensure that a single variant definition is used for submission to population and variant databases, aiding the filtering and interpretation of SVs by allele frequency.

This study identified two individuals recruited to the 100KGP who were compound heterozygotes for the NC_000015.10: g.28017720_28020677delinsTTT SV in *OCA2* and an additional pathogenic *OCA2* variant (Table 1). The *OCA2* SV deletes exon 7, resulting in a frameshift that leads to a premature termination codon in exon 8, with the aberrant transcript predicted to be eliminated by nonsense-mediated decay. Both carriers were of African or Afro-Caribbean ancestry and presented with a confirmed or suspected phenotype of FH but without reports of the hypopigmentation normally associated with OCA. The absence of such a conspicuous feature may reflect relatively poor phenotypic data available through the 100KGP, which may only disclose part of the full clinical manifestation. The selective and non-uniform reporting of phenotypes in the 100KGP has been discussed elsewhere [24].

Participants recruited to the 100KGP are predominantly of Caucasian origin, with only 3.1% (2767/90173) being of African ancestry (Data Release v19). However, across the entire 100KGP rare disease cohort, regardless of diagnosis, NC_000015.10: g.28017720_28020677delinsTTT was identified in 24 participants of African/Afro-Caribbean or mixed African/Afro-Caribbean ethnicity and only one of European ethnicity. Haplotype analysis of four carriers suggests NC_000015.10: g.28017720_28020677delinsTTT represents an African founder SV allele. A remarkably similar founder variant, a 2.7 kb deletion that eliminates exon 7 of the *OCA2* gene, was identified 30 years ago using PCR or southern blotting and was found in 78% (114/146) of 146 South African patients with OCA [31]. The primers used in previous studies [32] to detect this 2.7 kb deletion also span the NC_000015.10: g.28017720_28020677delinsTTT variant (Supplementary Fig. S2), making it highly likely that the previously reported 2.7 kb deletion is the NC_000015.10: g.28017720_28020677delinsTTT (which is 2958 bp in length) deletion described in this study. Enrichment of this SV in people of African origin has significant implications for diagnostic genetic screening in OCA. Individuals of African descent presenting with OCA [33] should be considered for an initial targeted assessment of the NC_000015.10: g.28017720_28020677delinsTTT variant before broader screening by WES or WGS.

In summary, we analysed previously unsolved FH cases using SVRare, an analysis package that annotates SVs with allele frequency data from the study cohort and/or other SV databases, the genomic context, regions of apparent CNV detected by Canvas and discordant pair and split-read alignments detected by Manta, to highlight potentially pathogenic variants. This approach solved 2/10 previously unsolved FH cases and identified the likely cause of FH-related disease in eleven additional unsolved cases from the 100 KGP. Furthermore, this analysis has identified multiple carriers of an *OCA2* insertion-deletion variant, which is almost certainly the same founder variant identified 30 years ago in OCA cases in Africa. This observation highlights inconsistencies in the reporting of SVs and reaffirms the need for long-range PCR and long-read sequencing to characterise SVs more accurately and ensure consistent reporting.

These results show that SVRare, in association with accurate clinical diagnoses, is effective in highlighting deleterious SVs, one of the major challenges in the diagnostic analysis of WES and WGS datasets. While financial and technical constraints impact the design of an optimal workflow, it is apparent that the ability to detect structural variants (and particularly variant breakpoints) is much improved using a WGS assay. As diagnostic centres begin to adopt long-read WGS as a first-line test [34], optimising the bioinformatics pipelines of third-generation sequencers by incorporating SVRare or similar software into the analysis pipeline, in combination with accurate clinical phenotyping, will be critical to achieving improved test sensitivity in the coming years.

## Supplementary information

Supplementary figures and table.

## Data Availability

Data from the National Genomic Research Library (NGRL) used in this research are available within the secure Genomics England Research Environment [35]. Access to NGRL data is restricted to adhere to consent requirements and protect participant privacy. SVRare data used in this research is available on (re_gecip/shared_allGeCIPs/JingYu-SV-query/protein_coding_genes_2021-11-170). Access to NGRL data is provided to approved researchers who are members of the Genomics England Research Network, subject to institutional access agreements and research project approval under participant-led governance. Structural variants identified were uploaded to the relevant LOVD databases: https://databases.lovd.nl/shared/genes/SLC38A8, https://databases.lovd.nl/shared/genes/PAX6, https://databases.lovd.nl/shared/genes/OCA2, https://databases.lovd.nl/shared/genes/GPR143 and https://databases.lovd.nl/shared/genes/CACNA1F.
