# Supplementary material for: Diagnostic discovery of structural variants causing foveal hypoplasia using SVRare and long-read nanopore sequencing
Source: Eur J Hum Genet. 2026 Jul 20;34(10):1493–502. doi: 10.1038/s41431-026-02190-y (PMC13634048; doi:10.1038/s41431-026-02190-y)
Supplement: Supplementary file 1 [file 41431_2026_2190_MOESM1_ESM.pdf]

NC_000015.10:g.27823041_27849013del

P3

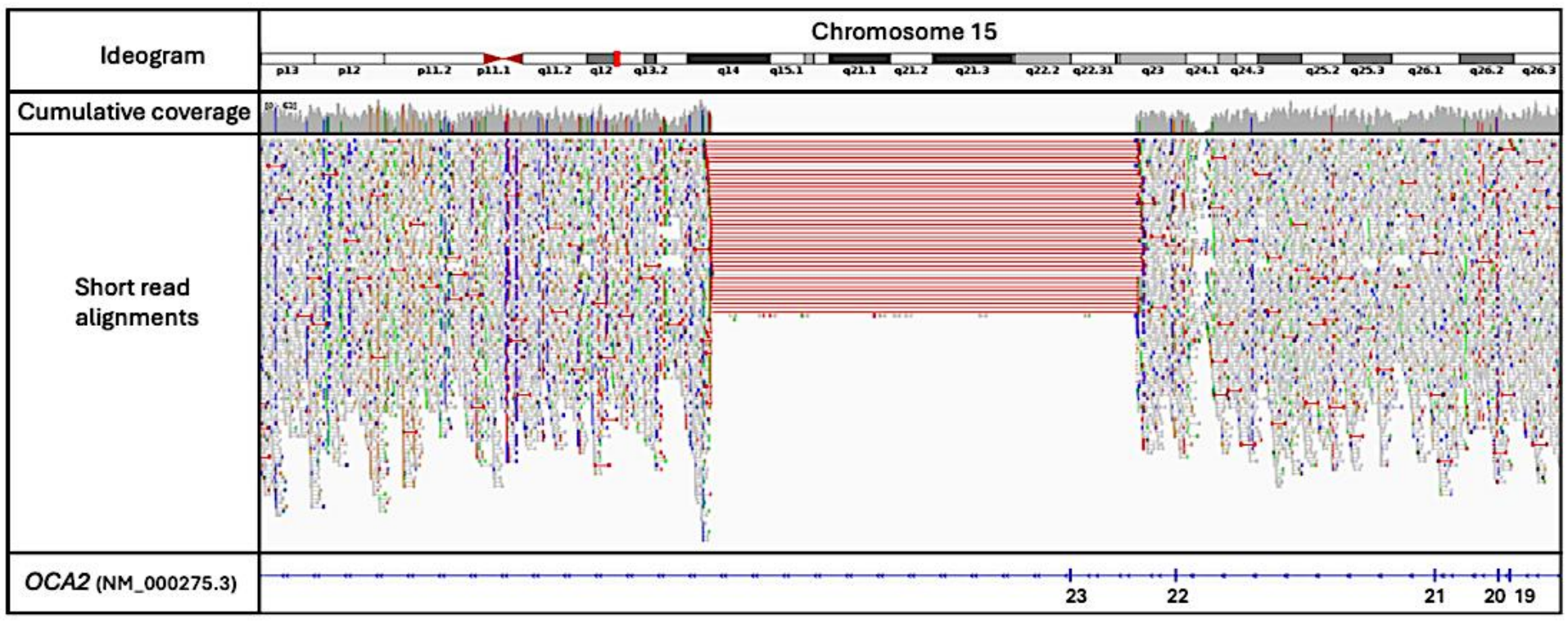

NC_000015.10:g.28017719_28020673del

P4

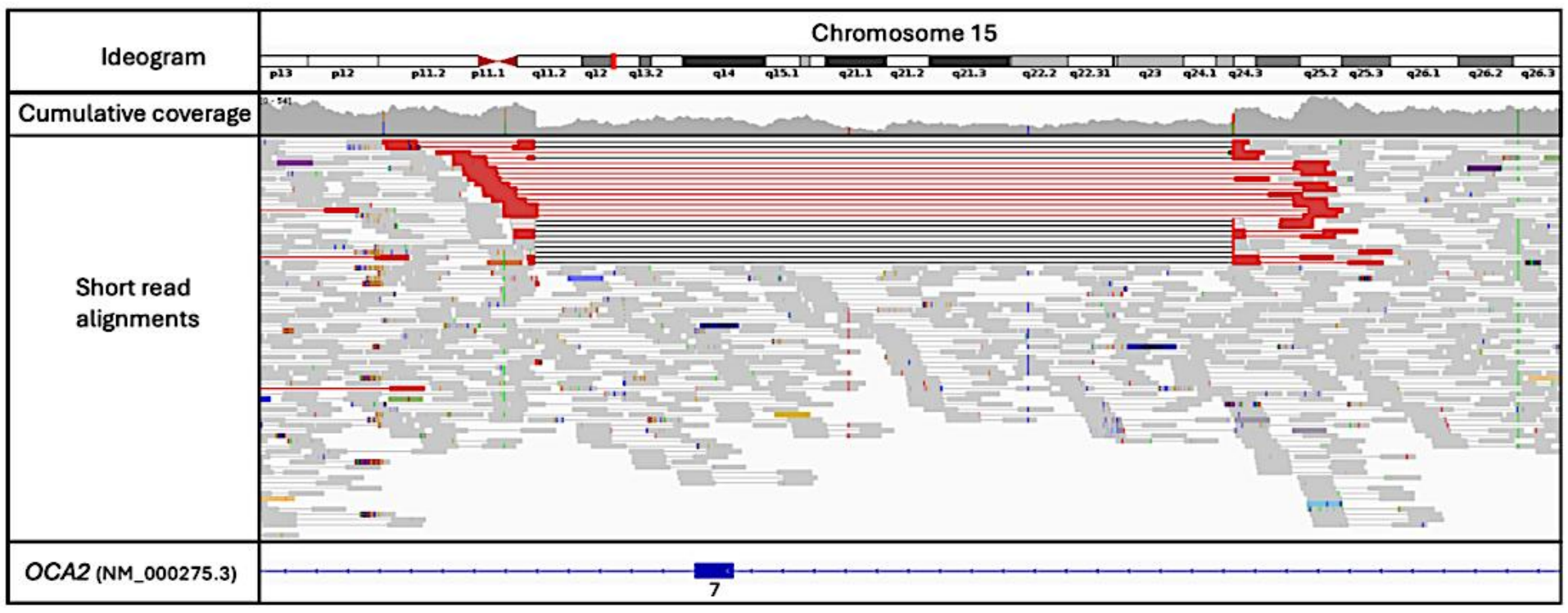

NC_000015.10:g.28017719_28020673del

P5

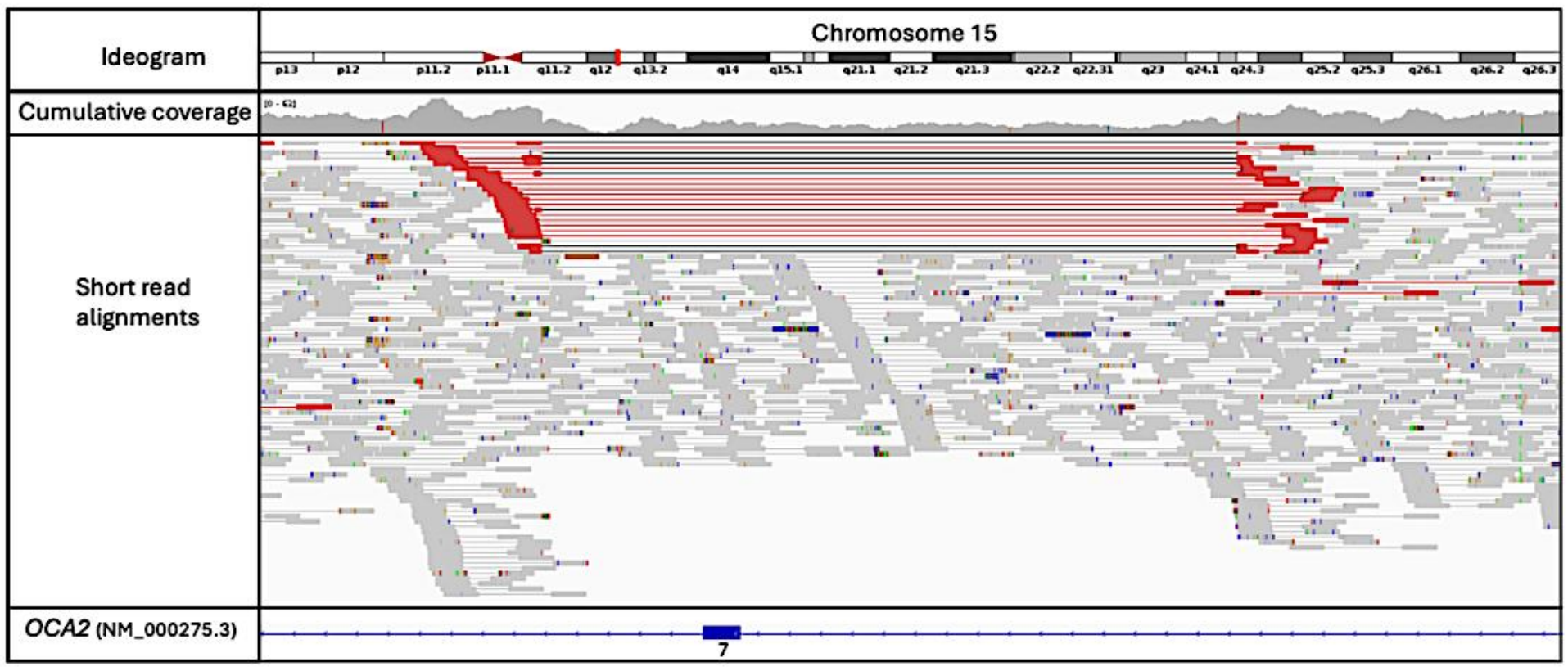

NC_000023.11:g.9476905_9954651del

P6

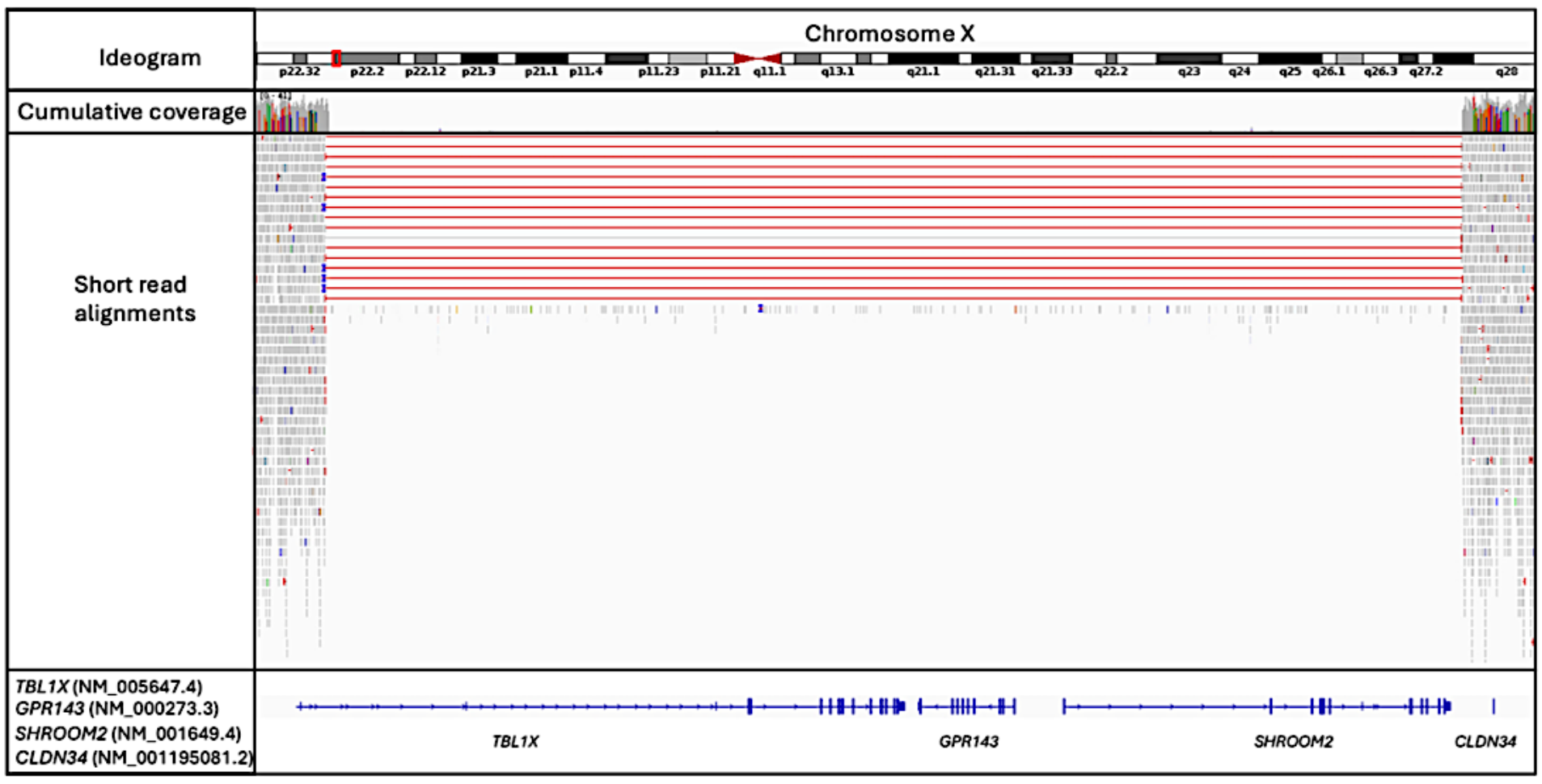

NC_000023.11:g.9476905_9954651del

P7

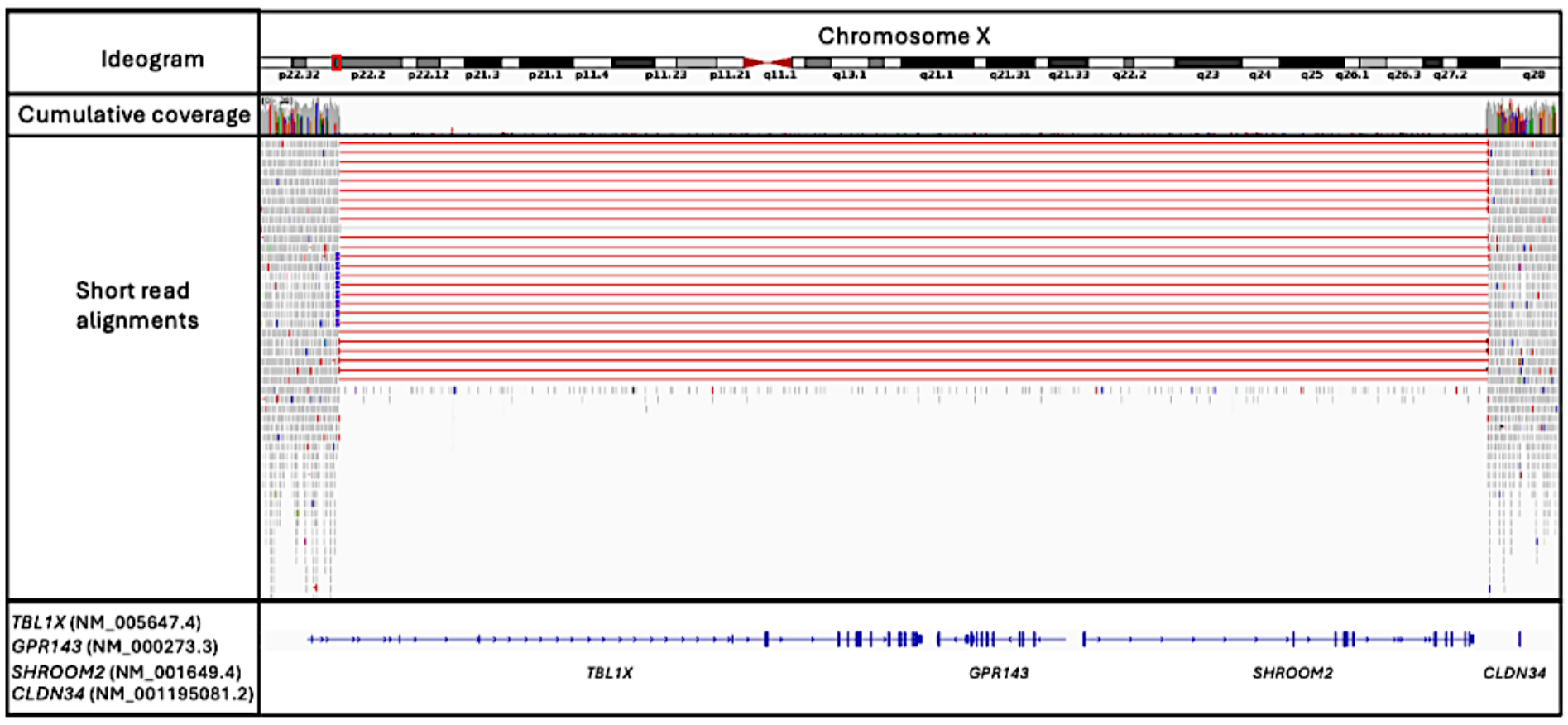

NC_000023.11:g.9743119_10024473del

P8

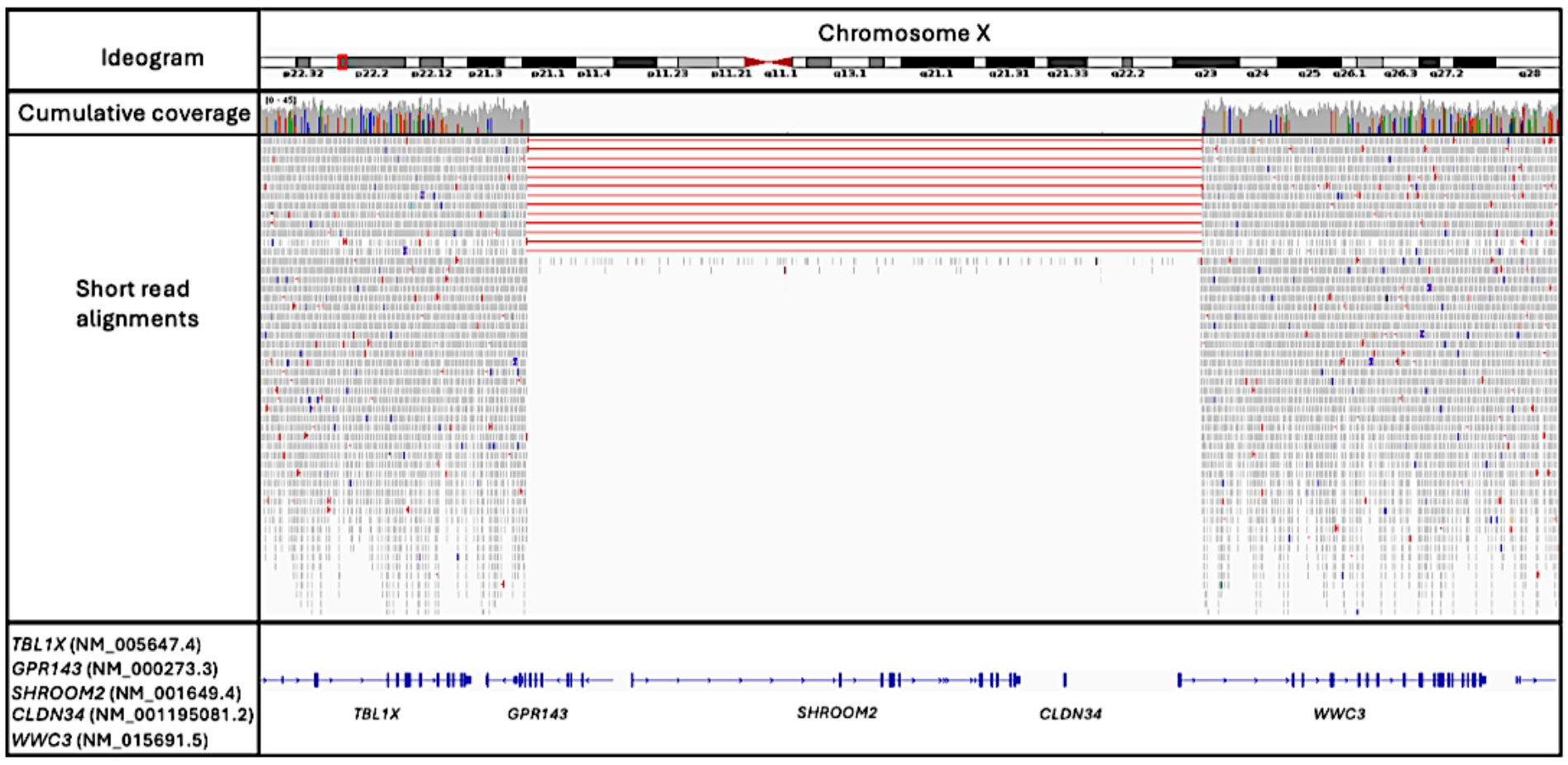

NC_000023.11:g.9760253_9761254del

P9

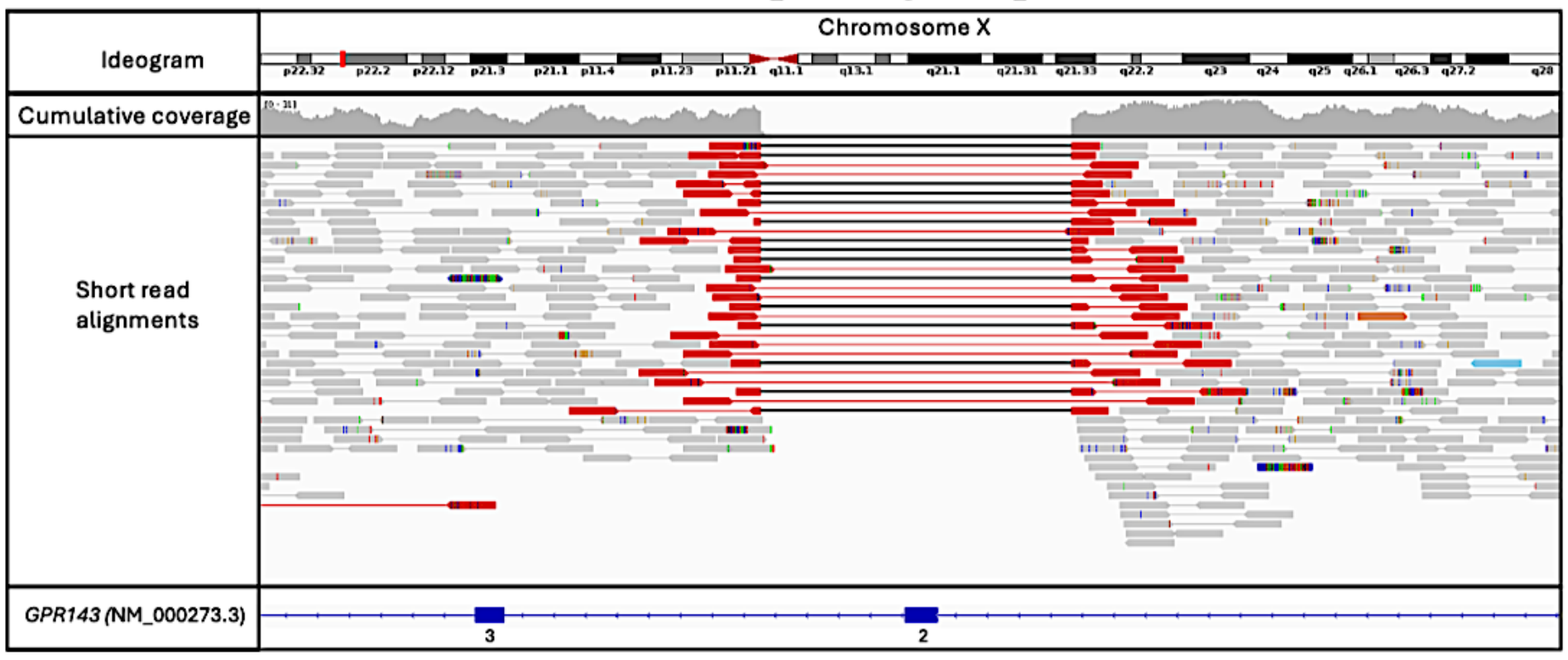

NC_000011.10:g.31393557_31812922del

P10

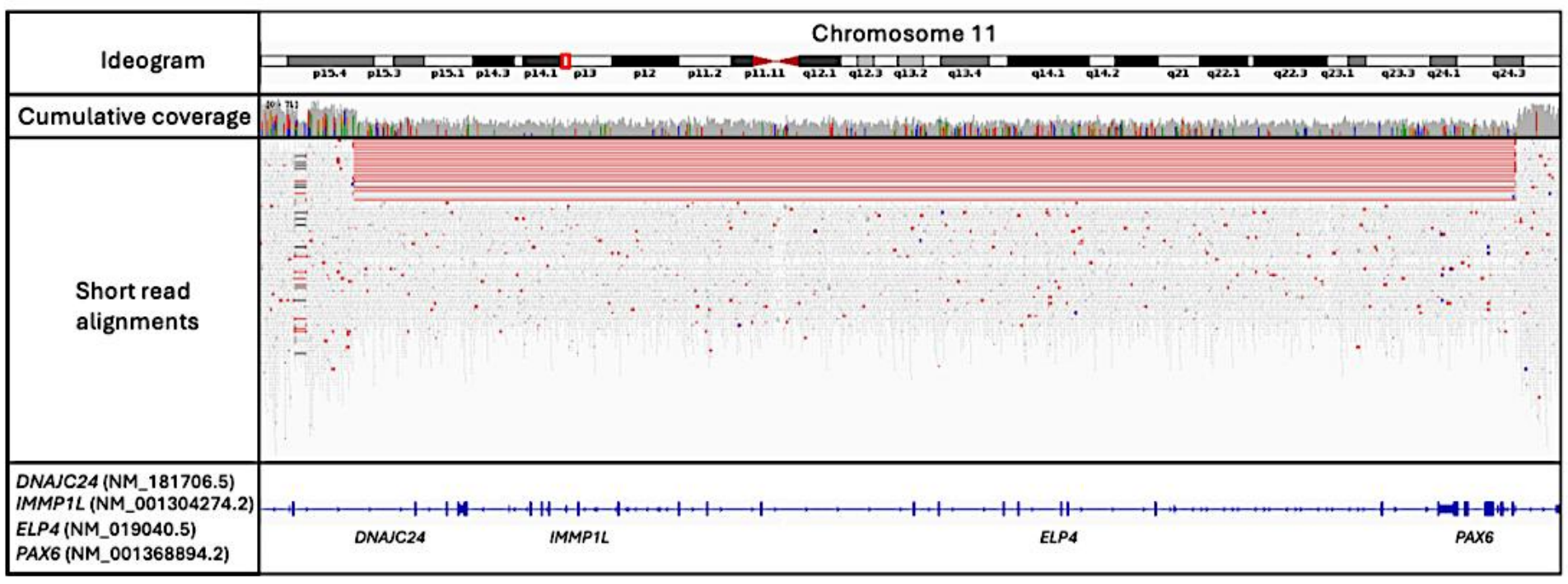

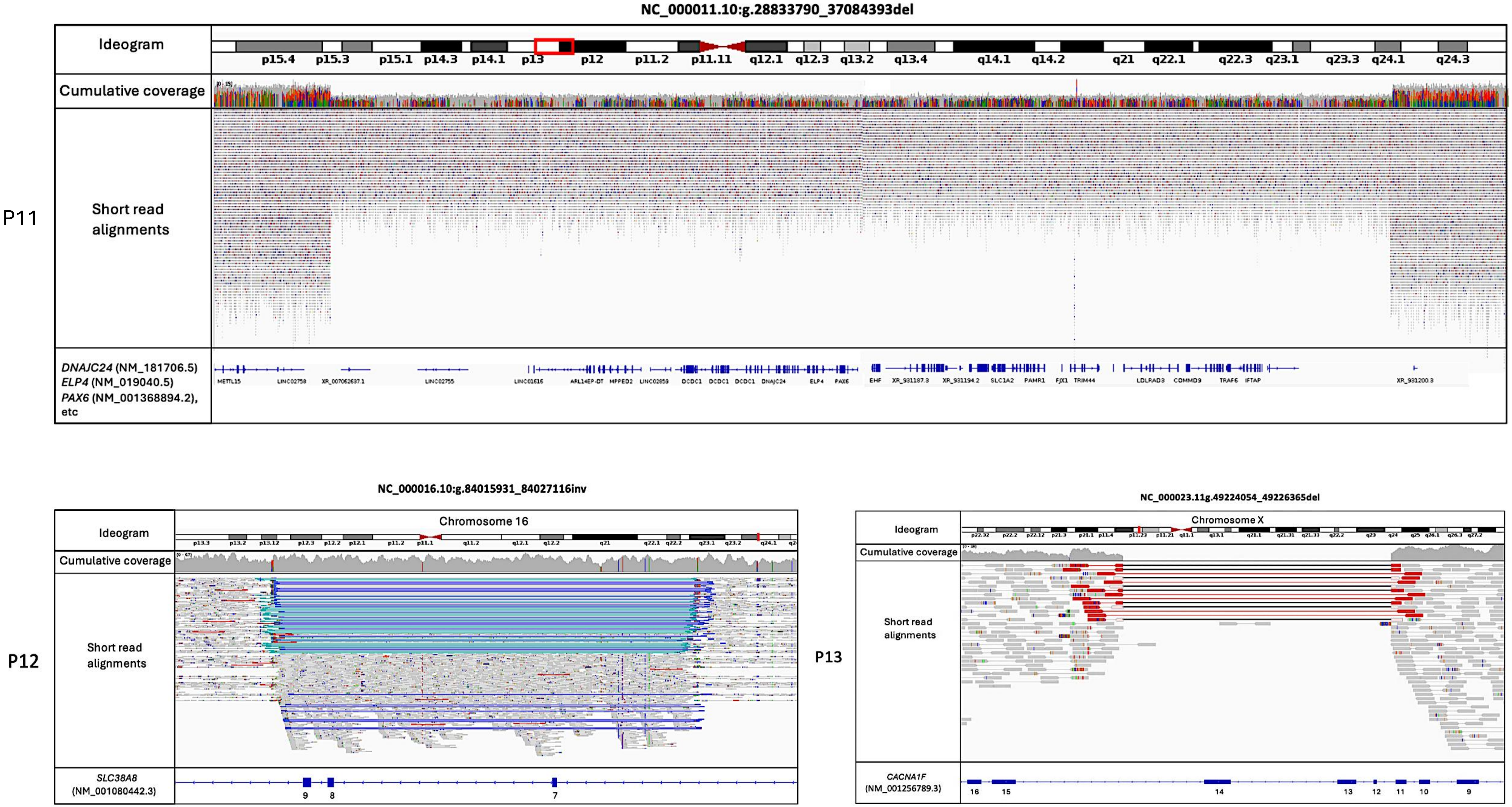

Supplementary Figure S1. Validation of SVs identified in the 100KGP. BAM file displaying the SVs at the target loci. Sashimi plot at the top of each figure represents the coverage across the genomic region inspected. The total read count is provided and the gene schematic is present at the bottom.

GRCh38	Reference	Family 1 Carrier 1	Family 1 Father	Family 1 Mother	Family 2 Carrier 2	Family 2 Father	Family 2 Mother	Family 3 Carrier 3	Family 3 Father	Family 3 Mother	African AF	dbSNP
15:27979889	G/G	T/T	T/T	T/T	T/T	T/T	T/T	T/T	T/T	T/T	0.9719	rs11074315
15:27980004	G/G	G/A	G/G	A/G	A/G	G/A	G/G	G/A	A/G	G/G	0.04376	rs59967422
15:27992851	A/A	G/G	G/G	G/G	G/G	G/G	G/G	G/G	G/G	G/G	0.9982	rs4499192
15:27994398	C/C	C/A	C/C	C/A	A/C	C/A	C/C	C/A	A/C	C/C	0.1626	rs57376758
15:27997088	A/A	A/A	A/G	A/A	A/AAGAAAGAAAG	A/A	A/AAGAAAGAAAG	AGAAAGAAAG/A	A/A	A/AGAAAGAAAG	0.1714	rs368785616
15:27999839	G/G	G/A	G/G	G/A	A/G	A/G	G/G	G/A	A/G	G/G	0.128	rs12101660
15:27999998	C/C	A/A	A/A	A/A	A/A	A/A	A/A	A/A	A/A	A/A	0.9999	rs4640132
15:28000188	G/G	G/G	G/G	G/G	G/A	G/G	A/G	G/G	G/G	A/G	0.3992	rs28818582
15:28000414	A/A	A/A	A/A	A/A	A/A	A/A	A/A	G/A	A/A	G/A	0.01754	rs149366789
15:28000665	A/A	A/A	A/A	A/A	A/G	A/A	A/G	A/A	A/A	A/A	0.1545	rs58913900
15:28002351	G/G	G/G	G/G	G/G	G/A	G/G	A/G	G/G	G/G	G/G	0.1534	rs74005195
15:28002374	C/C	C/C	C/C	C/C	C/T	C/C	C/T	C/C	C/C	C/C	0.135	rs74005197
15:28003579	C/C	A/C	A/A	C/C	C/C	C/C	C/C	C/C	C/C	C/C	0.1071	rs16950781
15:28003682	T/T	T/T	T/T	T/T	T/T	C/T	C/T	C/T	T/T	C/T	0.157	rs57242412
15:28005010	T/T	C/T	C/C	T/C	T/T	C/T	C/T	C/T	T/T	C/T	0.309	rs4778136
15:28011531	G/G	GA/G	GA/GA	G/G	G/G	G/G	G/G	G/G	G/G	G/G	0.1091	rs112437786
15:28011881	C/C	C/C	C/C	C/C	C/C	C/C	C/C	T/C	C/C	T/C	0.01654	rs143157494
15:28013609	C/C	C/C	C/C	C/C	C/T	C/C	T/C	C/C	C/C	C/C	0.06956	rs74005198
15:28014717	A/A	A/A	A/A	A/A	A/A	A/G	A/G	G/A	A/A	G/A	0.2194	rs4640131
15:28014907	G/G	G/A	G/G	A/G	A/G	A/G	G/G	G/A	A/G	G/G	0.1373	rs1800401
15:28019364	C/C	C/del	C/C	A/del	del/C	del/C	C/C	A/del	del/C	A/C	0.02032	rs112638567
15:28019708	A/A	A/del	A/A	G/del	del/A	del/G	A/G	G/del	del/G	G/G	0.4398	rs2871875
15:28020616	G/G	G/del	G/G	G/del	del/A	del/G	A/G	G/del	del/G	G/G	0.02916	rs78145457
15:28020856	G/G	G/G	G/G	G/G	G/G	G/G	G/G	A/G	A/G	A/G	0.08265	rs77490758
15:28021089	T/T	T/T	T/T	T/T	T/T	C/T	C/T	C/T	C/T	C/T	0.3672	rs746861
15:28021139	A/A	A/A	A/A	A/A	A/A	A/A	A/A	G/A	G/A	G/A	0.08184	rs74531804
15:28021881	G/G	A/A	A/A	A/A	A/A	A/G	A/G	G/A	A/A	G/G	0.3221	rs2122005
15:28022387	G/G	GTGGGTGAGAA/GTGGGTGAGAA	GTGGGTGAGAA/GTGGGTGAGAA	GTGGGTGAGAA/GTGGGTGAGAA	GTGGGTGAGAA/GTGGGTGAGAA	GTGGGTGAGAA/GTGGGTGAGAA	GTGGGTGAGAA/GTGGGTGAGAA	GTGGGTGAGAA/GTGGGTGAGAA	GTGGGTGAGAA/G	GTGGGTGAGAA/GTGGGTGAGAA	0.8557	rs11283428
15:28022612	A/A	G/A	A/G	A/A	A/A	A/A	A/A	A/A	A/A	A/A	0.08737	rs41534647
15:28022823	A/A	C/A	A/C	A/A	A/A	A/A	A/A	A/A	A/A	A/A	0.0134	rs148755931
15:28022866	T/T	T/T	T/T	T/T	T/T	T/T	T/T	C/T	T/T	C/T	0.01773	rs114176032
15:28023490	G/G	C/G	C/C	G/G	C/G	C/G	C/G	G/G	G/G	G/G	0.3323	rs7174197
15:28023844	C/C	C/C	C/C	C/A	C/C	C/C	A/C	A/C	C/C	A/C	0.2137	rs749846
15:28023862	A/A	C/C	C/C	C/C	C/C	C/C	C/C	C/C	C/C	C/A	0.8548	rs3794606
15:28024696	C/C	T/C	T/T	C/C	C/T	C/T	C/T	C/C	C/C	C/C	0.3327	rs28546555

Supplementary Table S1. Haplotype analysis in three families of African descent. Maternal alleles highlighted in orange and paternal alleles in blue. Uninformative alleles that cannot be phased in the carrier are in black. The "/" separates alleles on the homologous chromosomes. The red box encompasses the region deleted by NC_000015.10: g.28017720_28020677delinsTTT.

GGAGGGTGCATTCATTCTTCAGATGCTCCACTGCAGGTGACACAGCCTGTGCATGGGCGGGGTTTCCCTCTACTGGTTATAACAGGGAACATC
CATGAACCAGGGCTACGACTTCCATGCAGAAGTCACCGTGATAATCCAGTGGAGTCTCCGAGGCCAGGGCTCGGGGGAGTCAGGCTTGCG
TGGAAGGAGCCCTGCCTCTGGAGTCTAGGCTCTGGGCTGCCATGACAGCCACCGCTTCCTGGCTGCCTTTGCAGGATGTGAATGAAGGCTT
GACAAGGCTCAGAGACAAGGCTGAGCACAGGCCTACACAGATACAGCAGCTCAGAACGTAGGCACTGTCTTAGCAGATGTCCACAAACACA
GTAACCGTGCTGTGGAAGTGTCTTCTGAACCCTGACGTAAGGCCCGGACTTAACTGCACTTCAACGACACCAAAAAAAAAAAAAAAAGTTTTTT
CATTTCAAATGTATGTTTCCTGAATGGCCCAGCAAAACAGTTTTGAATAAAGAACTTGCAAGCCTGATTTTACTGAAATGGCAAGAGTTTCATAAAT
AGAGAAATGGTAAAAAGAGAATTTCAAAGACAAAGCAAAAGATAAGAAGAGCCAATGAATTGACTAAGAATGGTGTCCTCGCCTGTGGCTCCC
CATCAAATCCATTCAAGAGGAAACACAATTTATTATGAGATGAAATGAGATTTCACAATTCCTTTCAAATAAATTATCAGCATAACCTGCTGTGGCC
GCCGCCACCTGGAGCCCAAAGCGTCAGCCTGGGTCAGCTCCACCACGATGTGCTCTTCCCTCCCAGGACGACTCGGCCCACTGGCCACT
AGGGCCCCTGCCAGGTCCACCTGCAGCAGCGTGGAGTCCACGTGGCTGCTAAGGTTCACGGCTCGGAGAGTGTCAAGGAGAACCACAAG
GCAGACAGGAGAAACCCCATGTCCCCGCCGCCCGCCAGACGCACACCCTCCTCTGACAGTGTGTGAAGAAATGCGTTTCCCCAGGTGCC
CCACGCCCTTGGCCATTAACACGATCTTCCTGCCTTTCTTATTACCCACATCTCAGCCCTCATTCAAGACCTAACTCCCCTGAAGCAGATCGTC
GTGACCCTCTTGTGAGAGGTGCCCCCCACACCAGGGAAAGGGTGCATCCTTAGCTCCAAAGATGGAGGGAGGCCAAGAGGGACCTGAATG
GACAGGCCTCGCCAGGGCCCCTCCCGGCCCGTTTCCTGCGCTTGCTCATGTGCTCTCGTCCTATCACACCTTTCAGGACCCCACTCTTCAT
CAAACCTAGCATCAAAACACCAAGCTCAGCTGTTTCTTGTCAAGCAACGCTTATATGAAATACATGAGTATGCCTTTTTCTTGTTAACCTGTCCTTC
GTGTCTAATCTGCAGGGCCCTGGTTGGAGAAGCTAGGAGGGTGGGGAAACAGTGTTTTCTCCTCCCCTCTGAATCTCACAGTCTCCTCCATGT
CCTCTGAGAGCCCAGGATGCAGCGCTGGGTGTGATACAAAATGCATCACATGGGAAAAAGGAGAGAATGAAAGGGATGGGGAAAGAGGGA
GGGAGAGAATAGGGAGAATGGGGAGAAGGGAGGGAGAGAGAAAGGGAGAGGAGAAAGAAGAGGACGCAGCAAAGAAGAGCAGGAAGGG
AGGGAGGAAGAGAAAGGAGAGCGGGAGACAAGGACGGCGCCCTTAACTGACCTGGTTGTAGAGGTTTGCTGGCTTCTGTTCATTTGCCCAG
TGCCCTGGGAGCCTGACAGGCCTCCTTCAGACAACAATTCAGGGACAAAGAACTTCAGGTGCTACAATTACAGGCTTCTGAAATAGACCCTG
CAACTACCGAACCGACACCGCCTGGCTGCTGCGGGAGACACTGAGGTTCCGAAAGAAAGGTGGCTTCGCTATTCACCGGCTAAAGGAGAG
TGGTGCTTTCTCCAGAAGCTCCTCCCCTCACAGCAGACACCCTGGGTGTCCTTCACCCAGCCTCCTCCACAGGGCAGGCCAGAGGGCCG
GGAGGAGGCCTCGAGCTCCTTGCTCAGAAGACAGCCCTGAGGACATTGCTGTGGGCCTTTCCAACCAGGCCGAGGCCCCTCCTACCTCA
CGCCTATACTGCACGCCCTTTCTCAGGGAATCACCTTGTGGGGGGCAGGGTCCAGCTGGCTTCCCCTCCTCCCCAGGCAGGCCTCAGGC
CTGCAGCTTTCACAAAGCTCAGCTTTCACAAAGAGAGGGTCCGTGAGTTTCCGGAAAGCGTGGTTAATCCCTGGGAACACTGCCCTTTTCCAA
GGAGCCTGCTGGAGGCTCAAATCAACCCCTCATCGTGGCTGGTGACCCACTGCGCTGGAGGACGCAGCACCCGTCTTCCCCCCTGCGGC
CTTCATCTCTCCCAGGCTCTCTCTCTCCCTCTTCATCCAAATTCGAAGGCCACACAGGGAGCAGGCGAGACAGCTCCCCGCCCCCACCCAT
CCCCACCCCCTGCCAAGAAAGGCAGGCTGCCCTGCCCCCTGCTGGGAGGGACTCAGGCTGTGGGGTCCTGCGTGGGGCCTGGTGCACT
CAGGCTGCTCGAGAAGGAGCTCGGCAAAATGCCTTAGAATAAAGGGGACACACTGGGGCCTGGGTGGGATGACCAGAGGGCTGAGGCTAT
CATCTCATCTTAGTGACCTAAGCTGCTGTGCTCGCCTGGGATCTCTACTGACTGATCAAGTTTGGTGCCAGTGTTCCCCGAAATCTCCCCCTCC
TGCCTGAGTCCCACTGAGGACGGTTTCTGGACAGGATCCAGCCTCCCCCTTCAGGTTCCTGGGTCAGCCTGGCAGCCGGTTGCTCTATCTC
CCCTTCCCCCTGCGGGAAAGTCCTGCCCAGGAGGGTGGCATGTCCTGGGAACTTCATGAGGGGGGCCAGGAACACAGGGCAAGTCTTGG
CTTTCTTCATCCTGGAGAAGTAATGCAGAGGTCGAGGCACAAGAATGAGAAACAAAGTCTGTCAACATCACACAGCTCTTCGGTTCTCTCACAT
GGAGATACTTTACAGCTCAGGCACGGTTTTAAAGTGATGAAGAAAATACAAACACGTGCACACGCACTCAGCTGACATCAGCACCGCCCTGA
CCCCAGTGGAAGTGGAGTGCTTCTAAAACTGTGGGTGCCTCCATCCACTGAGCAGAGGGAAGCACAAACTGTGACCAGTGTGCCCCCGGCT
CACCAGAAAGCCTCAAGTGCCAGGCCTTTCTGGAAGGCTGCTGGTGCCCTGCTGGGTAGGTCACTGGCTAAGGAGAGGGTGTCCTGGGAA
GATGTAGTGAGACTGCCAGAAGGACTACAAAAACCAAGACTATGCAACAGATTACAGCAACTGCAAGGAAAGCAAGCCAGTTTCCTAAAATTT
CAGTTCACTGAGGCTATCAAGTAAGTAGACTAAAGAAAAAAACACTTGCCATTTAGCTAACTGAATTAATTCCTTTTTAAATAATAATCATCAAAAAT
ATTTGCCTGATCAGAGCACTGCATCGGAGGGTCCCACCTCTCCAGGACCCTCCACGGATGCCCGCCGCACTAGCAGTACCGGGGGCTTCA
GGGGCCTTCATGCACAGACACCTTAATGACCATGTCCATTCCAAAATAGGCTCTGGACCCTGGCTGGAGAGCTGAGAAACAGCCCTCCCAC
CCTTTCACCCAGAAAAGGCTGGGCGGACATCAGTTAACCGCCCACTCCTGATCCCAGAATAAGGCTGCAGCAGAAACAACTCGCCTGAAAC
CTAGAGCAAA

Supplementary Figure S2. Sequence and primers flanking the identified deletion in *OCA2*. Genomic locus Chr15:28017662-28021422 annotated with exon 7 (blue) and sequence deleted by NC_000015.10: g.28017720_28020677delinsTTT (red). Three primers from a previous study used in genotyping patients for a recurrent 2.7 kb deletion are highlighted in yellow [32].
